# A randomized-controlled trial focusing on socio-economic status for promoting vegetable intake among adults using a web-based nutrition intervention programme: study protocol

**DOI:** 10.1186/s12889-016-3907-y

**Published:** 2017-01-13

**Authors:** Saki Nakamura, Takayo Inayama, Takashi Arao

**Affiliations:** 1 Department of Health Promotion Sciences, Graduate School of Human Health Sciences, Tokyo Metropolitan University, Minami-Osawa 1-1, Hachioji, Tokyo 192-0397 Japan; 2 Research Fellow of Japan Society for the Promotion of Science, Kojimachi Business Center Building, 5-3-1, Kojimachi, Chiyoda-ku, Tokyo, 102-0083 Japan; 3 Faculty of Sports Sciences, Waseda University, Mikajima 2-579-15, Tokorozawa, Saitama 359-1192 Japan

**Keywords:** Nutrition education, Web-based intervention, RCT, Income, Vegetable, Japanese, Adult

## Abstract

**Background:**

Web-based nutritional education programmes appear to be comparable to those delivered face-to-face. However, no existing web-based nutrition education or similar programme has yet been evaluated with consideration of socio-economic status. The objective of a nutritional education programme of promoting vegetable intake designed a randomized controlled trial (RCT) is to evaluate the results of intervention and to determine how socio-economic status influences the programme effects.

**Methods/Design:**

Participants will be randomly sampled individuals (aged 30–59) stratified according national population statistics for sex, age, and household income. Participants were consented to survey participation (*n* = 1500), and will be randomly divided into intervention and control groups. The intervention period is 5 weeks with one step of diet-related education per week. The main outcome of the programme is dietary behaviour as eating vegetable (350 g per day, five small bowl). To encourage behavioural changes, the programme contents are prepared using behavioural theories and techniques tailored to the assumed group stages of behavioural change. In the first step, we employ the health belief model to encourage a shift from the pre-contemplative to the contemplative phase; in the second and third steps, social cognitive theory is used to encourage transition to the preparatory phase; in the fourth step, social cognitive theory and strengthening social support are used to promote progression to the execution phase; finally, in the fifth step, strengthening social capital and social support are used to promote the shift to the maintenance phase. The baseline, post intervention and follow-up survey was assessed using a self-administered questionnaire. For process evaluation, we use five items relating to programme participation and satisfaction. A follow-up survey of participants will be carried out 3 months after intervention completion.

**Discussion:**

The fact that this study is an RCT with an established control group is a strong advantage. Information and communications technology is not limited by time or place. If we could show this web-based nutrition education programmes has a positive effect, it may be an appropriate tool for reaching individuals in lower socio-economic state.

**Trial registration:**

Current Controlled Trials UMIN-ICDR UMIN 000019376 (Registered October 16, 2015).

## Background

Appropriate vegetable intake appears to be effective for cancer prevention [1, 2] and is associated with reduced risks of cardiovascular disease [3, 4], obesity [5], and other lifestyle-related diseases. Health policy initiatives are promoting vegetable intake across all segments of the population worldwide. However, in practice, it has been widely reported that vegetable intake remains low among social disadvantaged groups in terms of household income and other indicators of socio-economic status [6, 7].

As part of efforts to promote vegetable intake among adults, several nutrition education programmes that have incorporated aspects of behavioural science theory have proven effective [8–10]. Web-based intervention programmes are of particular note, and several have been developed and verified outside of Japan [10–20]. Lauren et al. [17] compared the effects of face-to-face nutrition education with a web-based education, and found that changes in vegetable intake prompted by the web-based programmes were comparable with those achieved through the face-to-face programmes. Additionally, Bensley et al. [16] reported that provision of information through bulletins board increased vegetable intake by 0.2 serving while a web-based intervention increased intake by 0.6 serving.

Despite these promising findings, only the reports by Buller and Ball [13, 19] considered socio-economic status in relation to web-based nutrition education programmes, indicating an extreme paucity of research in this area. Buller et al. demonstrated that it is possible to implement web-based nutrition education programmes even in agricultural communities lacking an adequate web infrastructure. Of course, a remaining challenge is that their findings are limited to agricultural communities. There are no studies comparing the effects of different socioeconomic status.

In Japan, the recommended daily amount of vegetables is 350 g, from the perspective of preventing lifestyle-related diseases [21]. Nevertheless, approximately two-thirds of adults do not meet this recommendation [22] – in particular, the average daily vegetable intake for adults is around 70 g below the recommended amount. The average daily intake among lower-income social groups is a further 70 g lower than that among higher-income groups. Our nationwide survey of adults on the actual situation regarding the relationship between socio-economic status and dietary habits showed that, lower-income groups are less likely to have a habit of eating five servings of vegetables daily than are higher-income groups [23]. However, even among higher-income groups, the proportion of individuals who customarily consume five servings of vegetables daily is extremely small, at approximately 10% of the population [24]. Thus, support for the increased intake of appropriate vegetables should be implemented using a population-based approach that targets not only lower-income groups, but also the entire adult population. We hypothesize that achieving an approximately 70 g (1 serving) increase in vegetable intake might help lower-income groups to catch up while simultaneously contributing to the partial resolution of the deficient vegetable intake amongst Japan’s adult population.

In Japan, Ministry of Internal Affairs and Communications report on Internet usage by Japanese citizens [25] shows that, today, the proportion of Internet users among all Japanese aged 30–59 is 90% or more. Furthermore, the proportion of lower-income individuals (i.e., who are earning less than two million JPY per annum; approx. 15,800 GBP) is 61%, and rising annually. A relatively high proportion of respondents to this report indicated that they use the Internet to search for information about health and medicine, ranging from 73% among 30-year-olds to 80% among 59-year-olds. The proportion of respondents who reported using the Internet at least once a week was as high as 91%. Thus, as a form of information and communications technology (ICT), the Internet is a powerful health education tool for which future expansion can be anticipated in fields of health promotion, especially nutrition [26]. Web-based nutrition education programmes by their nature rely on ICT and the Internet in particular [27]. However, in the context of web-based support for increased vegetable intake, there are almost no reports, even at the worldwide level, verifying the effects of these programmes according to income level.

In this paper, we describe the methods and protocol of a randomized controlled trial (RCT) that seeks to verify the effectiveness of a web-based nutrition education programme of promoting vegetable intake among adults and that is based on behavioural science theory. The main objective of the RCT is to develop and verify the effects of a 5-week programme of promoting vegetable intake. A secondary objective is to determine how the effects of this web-based nutrition education programme differ according to socio-economic status.

## Methods

### Study design

**Fig. 1:**
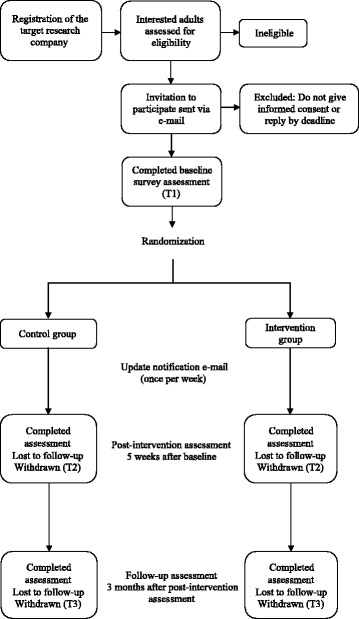
Flow chart showing participant recruitment, randomization, and evaluation of the Diet and Exercise Practices Project study

This study describes a two-armed, matched-design, web-based RCT. The nutrition education programme that we have developed as part of a health promotion project (Fig. 1). The intervention period is 5 weeks. The RCT is designed in line with the CONSORT statement for randomised trials of non-pharmacologic treatment [28]. Participants were assessed at three points in time: at baseline (T1), at post intervention (5 weeks later; T2), and at a follow-up at 3 months (T3). The participants were randomly assigned to one of two conditions: the intervention group and the waiting list group (i.e., the control group).

### Ethics approval

The RCT was conducted according to guidelines laid out in the Declaration of Helsinki for procedures involving human subjects, and has been approved by the Ethics Review Committee on Research with Human Subjects of Waseda University, Japan (2015-167). The respondent's privacy and personal information are fully protected due to agreement between the registration monitor and the social research company used to recruit participants. All e-mails were sent to participants by the research service company, and we have received a database containing only non-specific, anonymized data.

### Study sample

#### Recruitment source and procedure

A web-based intervention survey was conducted by a Japanese online research service company that contains data, including sociodemographic attributes, for approximately 160,300 adults aged 30–59 years. Participants were randomly selected based on Japanese population statistics for the present study. We targeted adults because of the necessity of health promotion, healthy eating, and reduction in health disparities for this age group [23]. The following inclusion criteria are considered: (1) men and women aged 30–59 years old; (2) able to understand Japanese; (3) can access the Internet at home, work, or a public place; (4) agree to access the study website during the 5-week intervention period; (5) will participate in all three assessment points during the 4-month study period. The exclusion criterion is having an household income of ten million yen or more (meaning that 88.4% of the total population is having an household income of ten million yen less) [29]. The study procedure from enrolment to follow up is depicted in Fig. 1.

The research company that we used in the present study periodically analyses and updates its registrant database. A notable characteristic of this company is that, to prevent bias among survey respondents, the firm requests the participation of the minimum number of respondents after taking response rate into consideration. Moreover, once every 6 months, the firm updates its monitoring information and conducts checks to safeguard against double registration or non-existent IP addresses. Registrants had been enrolled with the survey company by an open recruitment process.

Study participants were recruited using the following procedure. Of the approximately 160,300 registered monitors (as of September 2015), participants randomly to match the sex, age [30], and household income [29] distributions of Japan. Only study participants received an e-mail containing the website URL and password. Participate in the study were randomly assigned it to two groups in the order that received an answer to the intervention or control groups by the online research service company. The web-based nutrition educational programme is available in HTML format. Respondents who completed the questionnaire and clicked the ‘send’ button at the end of the online informed consent form were considered to have consented to survey participation (*n* = 1500). The research service company offered reward points valued at 40 JPY (in October 2015, one USD was equivalent to approximately 121 JPY) at T1. Of these, individuals who were allocated to the intervention group were offered reward points valued at 300 JPY after completion of the intervention (T2). Respondents from the control group were offered reward points valued at 40 JPY at T2. The participants were again offered reward points valued at 40 JPY for completing the assessment at 3 months after the completion intervention (T3; i.e. 4 months after the completion of the baseline survey [T1]).

### Setting

Regarding the setting, at baseline (T1), individuals consenting to participate in the study were randomly assigned it to two groups in the order that received an answer to the intervention or control groups by the online research service company. The researchers were not involved in this allocation in any way. All participants received a notification e-mail informing them of their allocated groups. The groups were listed as P Group (intervention group) and Q Group (control group) to prevent participants from knowing which group they were assigned to. This study is signal a type of single blinding. Because it is not the intervention of face to face, there is no contact during the intervention period.

### Intervention group

Participants assigned to the intervention group received an e-mail containing the dates of programme updates, the website URL of the intervention programme, a password for browsing the website, and the following information.

The programme updates were sent on Monday morning of each week during the intervention period. Participants received a total of five e-mails on the nutrition education programme over the 5 weeks of the intervention period. On the first occasion, participants received an e-mail containing (1) instructions on how to access the website (i.e. the URL and password), (2) an overview of the programme and how it would proceed, and (3) the programme contents for the first week. The e-mails sent in the subsequent weeks, in addition to item (1–3), included (4) a review of the contents of the previous step. The programme website was made freely accessible only for the duration of the intervention period, and after which it was closed. Participants’ passwords were effective for the duration of the study. If a participant forgot his or her password, it could be retrieved by contacting a research officer. After completion of the 5-week intervention, participants received an e-mailed request to participate in the post-intervention survey (T2). Finally, participants received another e-mail request to participate in a follow-up survey 3 months after completing the intervention (T3).

### Control group

The control group surveys took place over the same period as intervention group surveys. Control group participants received an advance notification e-mail after the baseline from the survey company that another survey would take place 5 weeks later. After a 5-week-long silence, participants received an e-mail request to take part in the post-intervention survey (T2). Subsequently, participants received an e-mail request to participate in a follow-up survey 3 months later (T3).

### Intervention programme

**Fig. 2:**
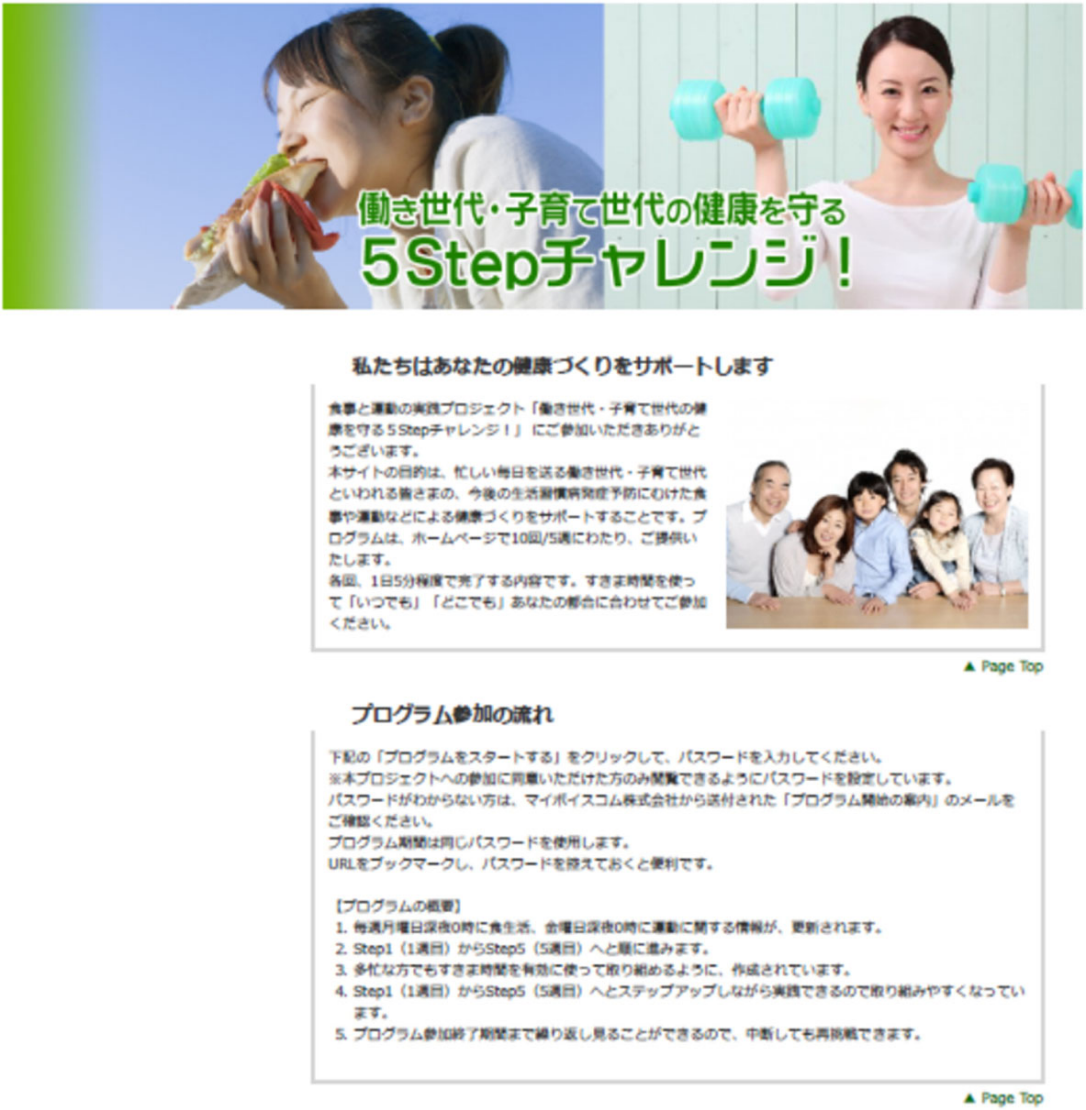
A snapshot of the website

An interactive webpage called the ‘Diet and Exercise Practices Project (http://healthpromotionqol.com)’ is designed to improve vegetable intake of visitors to help decrease the likelihood of lifestyle-related diseases (Fig. 2). It is a free website that provides information, a monitoring sheet, and advice about healthy diets, increasing vegetable intake, and preventing lifestyle-related diseases. We hypothesize that achieving an approximately 70 g (1 serving) increase in vegetable intake might help lower-income groups to catch up while simultaneously contributing to the partial resolution of the deficient vegetable intake amongst Japan’s adult population. Therefore, the action goals of intervention is a vegetable dish to eat five servings, or was a plus 1 serving (approximately 70 g) per day. The programme is divided into five steps that align with the stages of behaviour change. Table 1 shows the framework of the programme. To be updated every week, but watch the past step not proceed in the previous step. It was created to advance the based on behavioural science theory. To avoid contamination during the intervention period, access to the webpage was password-restricted so that only study participants could visit it.

**Table. 1:** Five steps and accompanying components of the dietary education intervention

Step	Stages of change and message	No	Items	Topics	Practice points (e.g., worksheets)	Behavioral science theory	Behavioral modification technique	Health literacy	Processes of change
1	Precontemplation → Contemplation (Encourage interest in vegetables)	1	Today's points	Eating a lot of vegetables is good for our health		Health belief model	Perceived susceptibility, perceived severity	Seeking information from various sources, Extracting relevant information	Dramatic relief, raising awareness
		2	Do you know?	It is recommended that we consume 350 g of vegetables per day	Points: 350 g of vegetables = 5 servings	Cognitive behavior theory	Cognitive restructuring	Seeking information from various sources, Considering the credibility of the information	Dramatic relief, raising awareness
		3	Easy to devise	Self-check! Counting the number of vegetable dishes	Self-check: How many vegetable dishes (servings) do you eat per day?	Social cognitive theory, theory of planned behavior	Self-monitoring	Considering the credibility of the information	Raising awareness
		4	Let's try it!	Live a healthy life by eating vegetables daily	Worksheet: record the number of vegetable dishes (servings) that you ate daily.	Social cognitive theory, theory of planned behavior	Subjective norm, self-monitoring	Considering the credibility of the information, Making decisions based on the information	Raising awareness
2	Contemplation → Preparation (Try to increase number of vegetable dishes by one (+1))	1	Today's points	You decide your one dish (+1)	Points: Let's choose the “ + 1” with the confidence so we can practice it at home and in restaurants.	Social cognitive theory, theory of planned behavior	Contingency management	Extracting relevant information	Self-reevaluation
		2	Do you know?	At home: Point the convenience easy recipes for your one dish (+1)		Social cognitive theory, theory of planned behavior	Role playing	Seeking information from various sources, Extracting relevant information	Raising awareness, counterconditioning
		3	Easy to devise	Eating out: How to choose simple dishes depending on your one dish (+1)		Social cognitive theory, theory of planned behavior	Role playing	Seeking information from various sources, Extracting relevant information	Raising awareness, counterconditioning
		4	Let's try it!	Let's do so! Eat your one dish (+1) from today onwards	Worksheets: I will try my “ + 1” for today's meal!	Stimulus-response theory, social cognitive theory	Stimulus control, goal setting, self-monitoring	Extracting relevant information, Making decisions based on the information	Self-liberation
3	Contemplation →Preparation (Meals can be opportunities to evaluate one's dietary balance)	1	Today's points	Eat a proper meal based on your confidence in the future of your health	Points: Let's check whether a meal contains everything (i.e., a staple food, a main dish, side dish) or not.	Social cognitive theory, theory of planned behavior	Contingency management, commitment	Extracting relevant information	Self-reevaluation
		2	Do you know?	At home: Tonight's dinner is now OK		Social cognitive theory, theory of planned behavior	Role playing	Seeking information from various sources, Extracting relevant information, Making decisions based on the information	Raising awareness, counterconditioning
		3	Easy to devise	Eating out: balance achieved by combining foods is OK	Try: Let's choose meal that contains a staple food, main dish, side dish when eating out or at a convenience store.	Social cognitive theory, theory of planned behavior	Role playing	Seeking information from various sources, Extracting relevant information, Making decisions based on the information	Raising awareness, counterconditioning
		4	Let's try it!	Challenging your to balance your meal	Work sheets: I practice the goal that this myself decided!	Social cognitive theory, theory of planned behavior	Role playing, commitment, self-monitoring, behavior analysis	Extracting relevant information, Understanding and communicating the information, Making decisions based on the information	Self-liberation
4	Preparation →Action (Precautions for a healthy diet)	1	Today's points	What do you do if you cannot eat?		Social cognitive theory, theory of planned behavior	Contingency management	Seeking information from various sources	Environmental reevaluation
		2	Do you know?	Practical examples: recommended measures		Stimulus-response theory	Stimulus control, counterconditioning	Extracting relevant information, Considering the credibility of the information, Making decisions based on the information	Stimulus control, counterconditioning
		3	Easy to devise	Characteristics of those who are eating plenty of vegetables	Self-check: What precautions do you take? Check each item that applies!	Stimulus-response theory	Counterconditioning, modeling, self-monitoring, behavior analysis	Seeking information from various sources, Considering the credibility of the information, Extracting relevant information	Stimulus control, counterconditioning
		4	Let's try it!	Devise a goal: Let's make precautions for when something does not work	Try: In case eating a “ + 1” does not go well, let's decide on a precaution beforehand.	Stimulus-response theory	Commitment, stimulus control	Extracting relevant information, Considering the credibility of the information, Making decisions based on the information	Stimulus control, self-liberation
5	Action →Maintenance (Healthy community helps us)	1	Today's points	Everyone's goal is to improve their health		Social cognitive theory, theory of planned behavior, social network, social support	Contingency management, self-monitoring	Extracting relevant information, Considering the credibility of the information, Making decisions based on the information	Helping relationships, social liberation
		2	Do you know?	Eat a delicious meal together with family and friends!	Self-check: What kind of topic do you talk about with family and friends?	Social cognitive theory, theory of planned behavior	Subjective norms, self-monitoring	Extracting relevant information, Understanding and communicating the information, Making decisions based on the information	Helping relationships, social liberation
		3	Easy to devise	Investigate safe diets in the community	Try: Let's look for a shop that supports health promotion in your community!	Social cognitive theory, theory of planned behavior	Subjective norms	Seeking information from various sources, Extracting relevant information	Helping relationships, social liberation
		4	Let's try it!	Let's be healthy by eating vegetables! Spend a day having a fun diet	Try: Let's review a past topic! Try: Let's settle on a “ + 1” that you can begin introducing to family and friends! Free description: Questions and free comments	Social cognitive theory, theory of planned behavior	Commitment, self-monitoring, contingency management	Extracting relevant information, Considering the credibility of the information, Understanding and communicating the information, Making decisions based on the information	Social liberation

In order to achieve this action goals were designed following the intervention programme. To encourage behavioural changes, the programme contents are prepared using behavioural theories and techniques tailored to the individual stages of behavioural change. In the first step, we employ the health belief model to encourage a shift from the pre-contemplative to the contemplative phase; in the second and third steps, social cognitive theory is used to encourage transition to the preparatory phase; in the fourth step, social cognitive theory and strengthening social support are used to promote progression to the execution phase; finally, in the fifth step, strengthening social capital and social support are used to promote the shift to the maintenance phase. All of the steps is composed of four items, as introducing “Today's point (Including review of the previous week from the second week onwards)”, information and skills necessary to behaviour modification as practical content “Do you know?” and “Easy in devising” are on two page, as summary “Let's try it !(to support behaviour change by using a work sheet)”.

### Web design

**Fig. 3:**
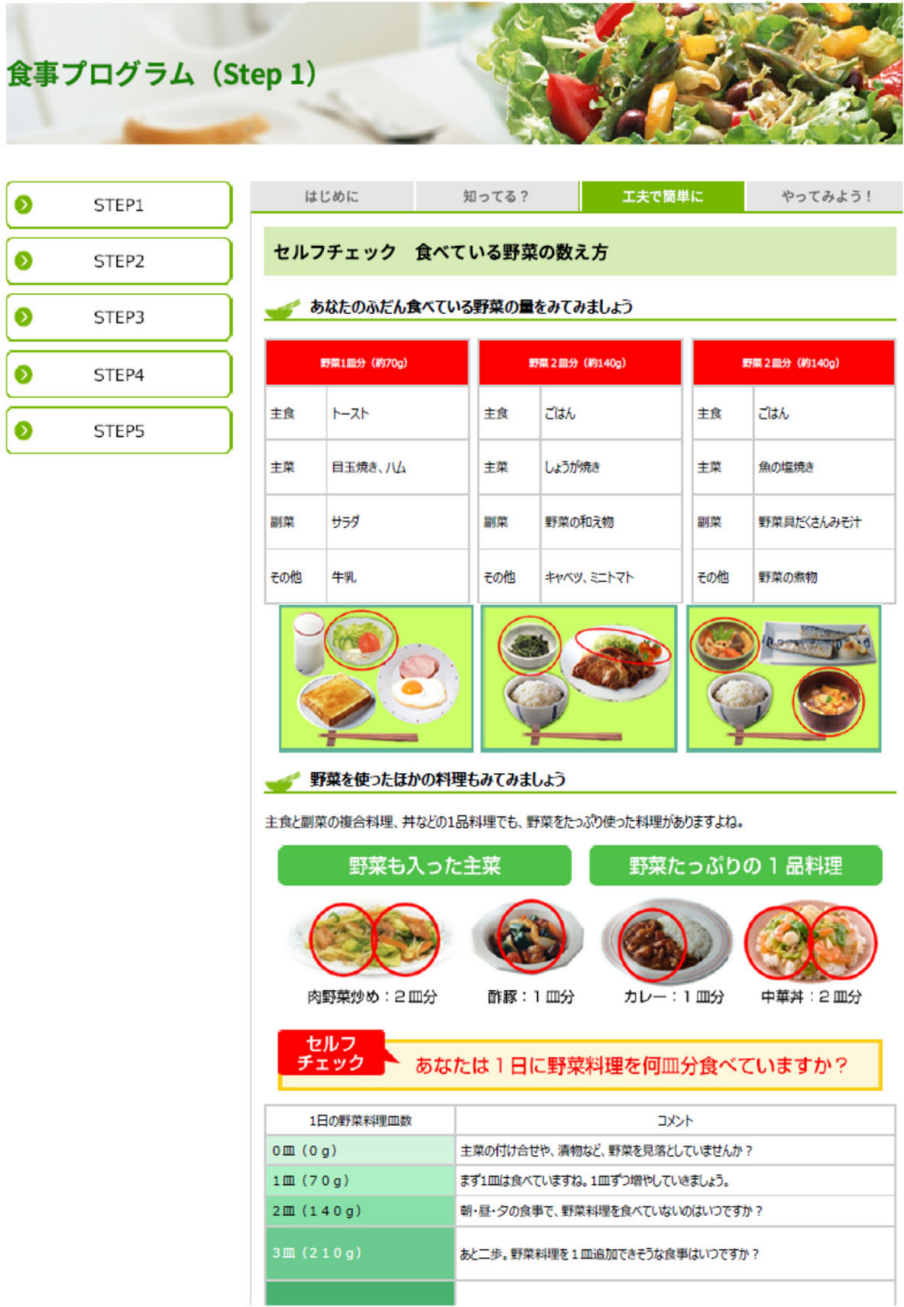
A snapshot on programme page of the website

**Fig. 4:**
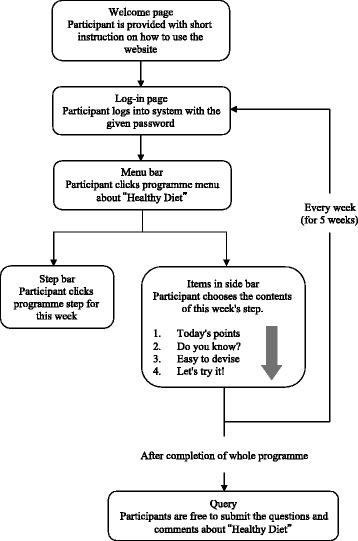
Website structure

The website design was settled on through consultations with registered dieticians, health movement educator, and public health experts. Regarding the size of the online text, the amount of information, and the configuration of the website, we obtained the advice of web design professionals. Figures 2 and 3 show a snapshot on programme of the website, while the website structure is shown in Fig. 4.

### Assessment

The primary outcome variable was assessed using a self-administered questionnaire in Japanese. Regarding the assessment, at baseline (T1), primary outcomes, transtheoretical model [31], self-efficacy [32], dietary knowledge [33], perceptions of neighborhood food environments within secondary outcomes, and all other measurements. After completion of the 5-week intervention, participants received an e-mailed request to participate in the post-intervention survey (T2). At post-intervention survey (T2), we assessed the all primary outcomes, all secondary outcomes, all other measurements excluding the subjective economic status. We evaluated about the participants’ satisfaction of process evaluation as after intervention. Finally, participants received another e-mail request to participate in a follow-up survey 3 months after completing the intervention (T3). At follow-up survey (T3), we assessed the all primary outcomes, all secondary outcomes, and all other measurements excluding the subjective economic status.

### Primary outcomes

As Health Japan 21 (the secondterm﻿), it is necessary to evaluate the intervention that applies to the improvement of diet-related quality of life. Interventions that focus on dietary lifestyle require evaluation of diet-related quality of life as primary outcomes. Two of the subscales of the Subjective Diet-Related quality of life scale includes dietary satisfaction and fun of meals. As primary outcomes, we considered Subjective Diet-Related quality of life, which comprised an assessment of the final dieting goal (2 items) [34], and self rated health, which has been reported to be associated with both socio-economic status and mortality [35, 36].

### Secondary outcomes

The secondary outcomes were eating behaviours, which was considered an index of behavioural change and is often held up as the goal of nutrition education. Three eating behaviours (per week) were considered: mealtime balance, or eating ‘balanced meals comprising a staple food, a main dish, and a side dish’ [37], ‘eating dark green vegetables’, and ‘eating full servings of vegetables (5 small dishes or approx. 350 g per diem)’ [38]. The mealtime balance behaviour has been shown to facilitate nutrient intake and improve nutritional status [39–41]. To explain the staple foods, main dishes, side dishes, and vegetable servings, we posted sample photographs for reference in terms of size and amount, and stipulated that respondents should always check these photographs before responding. Because daily eating behaviours (two items) were checked using these photographs depicting single (70 g) servings of vegetables, participants were also asked to answer how many vegetable servings they consumed per diem. This item reflects the fact that, as demonstrated by Ozawa et al. [42], the behavioural goal of ‘five or six small [vegetable] dishes a day’ can potentially serve as an easily grasped indicator of consuming 350 g per diem. Additionally, the amount of vegetables eaten each day (in g) was also self-reported.

### Other measurements

As our intention was to evaluate not only outcomes, but also the processes leading to behavioural change, we also employed secondary items assessing diet. We used a measure based on the transtheoretical model [31] (containing three items) to evaluate the intermediate factors relating to secondary-outcome eating behaviours, and a measure of self-efficacy [32] (also containing three items) as an evaluation of preparatory factors. We also used two items [33] to assess dietary knowledge concerning secondary-outcome eating behaviours. Perceptions of neighborhood food environments relating to eating behaviour was assessed with ten items [43, 44].

We also measured health literacy [45], which is defined as the knowledge, desire, and skills for acquiring, comprehending, evaluating, and making use of health information, using a scale with good validity for the Japanese population. In the context of our previous research, the relationship of health literacy with vegetable intake behaviour was shown to be unaffected by socio-economic status, whereas promotion of vegetable intake behaviour has the potential to improve health literacy [46]. In addition, we measured subjective economic status [47], household income [30], educational attainment [23, 24, 46], and other attributes (namely sex, age, marital status, living status, and employment status).

### Process evaluation

Adherence to the intervention was assessed by the number of log-ins and duration spent in the website. Furthermore, the participants’ satisfaction with the intervention was assessed by using a self-administered questionnaire at post-intervention. With reference to previous studies on programme development [48, 49], we also included an evaluation (5 items) of the nutrition education programme itself. The items were as follows: (1) was the content of the programme fun? (2) was the content of the programme easy to understand? (3) after participating, did you become aware of any problems in your own diet? (4) did you feel the programme was helpful as a health management material? and (5) as a health management material, did it make you want to participate again another time?

### Sample size

The sample size was calculated using G*Power [50]. We set an effect size of 0.5, an α of 0.05, and a power of 0.95. With the expectation that two-thirds of participants would drop out, the size of the intervention group was set at 900 subjects, while that of the control group was set at 600 subjects with the expectation that a half of participants would drop out. Furthermore, we aimed to divide the intervention and control groups further according to income (low and high).

### Statistical analyses

All statistical analyses will be performed with IBM SPSS Statistics 21.0. For continuous variables, independent t-tests will be used to determine inter-group differences and Man–Whitney U-tests for intra-group comparison, while the χ^2^ or an equivalent test is to be used to determine the associations between categorical variables. Because this is a prospective RCT involving repeated measures, repeated measures analyses of variance will be applied to determine significant differences within the study groups. We also considered the possible effect of time on groups. The evaluation of the intervention is based on an intention-to-treat analysis. A *p*-value of 0.05 has been set as the level of significance.

## Discussion

This study describes an RCT of a web-based nutrition education programme. Promoting vegetable intake is an important challenge for health promotion during adulthood, regardless of socio-economic status. Japanese adults of all household income levels consume less than approximately two-thirds of the recommended daily amount (i.e. 350 g or five servings per day). Support for the increased intake of appropriate vegetables seems best implemented using a population-based approach, as this issue is necessary for not only lower-income groups, but also the entire adult population. Web-based nutrition education programmes are communication tools that use ICT, in particular the Internet. In other words, it is a health education tool for which future expansion can be anticipated in fields of health promotion. If we could show that such web-based nutrition education programmes are beneficial for improving vegetable intake, our population-based approach would enable lower-income groups to catch up in terms of averaged daily intake of vegetables while also contributing to the resolution of the overall deficiency in vegetable intake in Japan’s overall adult population. So far as we know, this is the first report regarding a web-based nutrition education programme developed with a focus on socio-economic status.

### Strengths and limitations

This study has three main strengths. First, this is an RCT using high-quality research methods. Because the RCT participants were assigned randomly, there is very little allocation bias. Additionally, randomization took place after acquiring consent to participate, which means that the intervention group was not populated only with participants actively interested in the research topic. This helps in avoiding over-estimation of the effects of the nutrition education programme in the intervention group. Second, this is nutrition education programme was web-based, thus making it highly accessible. Furthermore, the study was carried out by a social research company with a wealth of experience in implementing academic research surveys. These points ensured high feasibility while minimizing the burden of securing an adequate sample size to increase statistical power. In addition, participants could be extracted to match the distribution of sex and age in the Basic Resident Register and household income in the Comprehensive Survey of Living Conditions. For this reason, possible confounding effects could for the most part be eliminated at the stage of allocation. Third, the programme affords numerous advantages for both participants and supporters. Participants can take part when they are free to do so (e.g. time, place, and scheduling). As this means that they can take part even if they are located far away from the researchers, it would be highly convenient for participants [26]. The programme also offers supporters the opportunity to provide a unified nutrition education programme that is not reliant on the number of participants and to provide support without being subject to spatial or geographical restrictions. While it is true that the initial cost is somewhat considerable, its operational costs are low. The economic burden on supporters is thus small. Furthermore, passwords can be set for browsing the nutrition education programme, which can prevent communication between groups that could impact the quality of research.

The RCT in this study is original in the following two regards. (1) It can verify the intervention effect for an adequate sample size, and (2) it can verify differences in the effect of a nutrition education programme by income strata. With this study, it will also be possible to verify the possible catch-up effect in relation to undesirable vegetable intake behaviour associated with low household income. Some reports have suggested that traditional classroom-based nutrition interventions might widen this disparity [6]. In contrast, with our study, it may be possible to show that a web-based nutrition education programme would inhibit the widening of this disparity.

Conversely, this study also has its limitations. It is possible that the results of the study will reflect the characteristics of individuals registered with the research company (e.g., tending to be young, high-earning, and highly educated [51–53]). To minimize this potential limitation, we have made sure to match participants with demographic distributions in terms of sex, age, and household income. In addition, we requested research cooperation from individuals whose household income data – which was our objective – had been recorded in advance. This helps to restrict attribute bias as well as negative response rates for delicate questions pertaining to household income and other matters. Second, during participant recruitment, we excluded individuals reporting household incomes of ten million JPY or more. This means that the results do not target the entire population. Accordingly, care must be taken when drawing general conclusions. However, a limit of less than ten million JPY still applies to 88.4% of the Japanese population. Finally, participants did not have contact with one another (either in person or through indirect communication) for the period of the intervention. For this reason, it is possible that rates of adherence to nutrition education programmes will be lower than they would with enforced participation.

### Implications for practice

The promotion of vegetable intake behaviour is an important challenge for health promotion during adulthood, regardless of socio-economic status. ‘Health Japan 21 (the second term)’ policy initiative [21] is promoting the use of ICT as a health promotion strategy for the future. Because ICT frees individuals from the limitations of time or place, it may be a tool that encompasses individuals in lower socio-economic strata who have little time to spare. If we can show that web-based nutrition education programmes have a positive effect, our study would represent the outcome of a population approach as a health promotion strategy.

## Abbreviations

ICT: Information and communications technology
RCT: Randomized controlled trial
